# Five-Minute Cognitive Test as A New Quick Screening of Cognitive Impairment in The Elderly

**DOI:** 10.14336/AD.2019.0115

**Published:** 2019-12-01

**Authors:** Jie Zhang, Lijun Wang, Xia Deng, Guoqiang Fei, Lirong Jin, Xiaoli Pan, Liuhan Cai, Anthony D Albano, Chunjiu Zhong

**Affiliations:** ^1^Department of Neurology, Zhongshan Hospital; State Key Laboratory of Medical Neurobiology; Institute of Brain Science; Fudan University, Shanghai, China.; ^2^Department of Psychometrics, Research, and Data, Measured Progress, Dover, NH 03820, USA; ^3^Department of Educational Psychology, University of Nebraska-Lincoln, Lincoln, NE 68588, USA

**Keywords:** mild cognitive impairment, Alzheimer’s disease, brief cognitive test, equipercentile equating method

## Abstract

This study aims to develop a new evaluation method for quickly and conveniently screening cognitive impairment in the elderly. The five-minute cognitive test (FCT) was designed to capture deficits in five domains of cognitive abilities, including episodic memory, language fluency, time orientation, visuospatial function, and executive function. Subsequently, FCT efficiencies in differentiating normally cognitive ability from cognitive impairment were explored and compared with that of the Mini-Mental Status Evaluation (MMSE). Equipercentile equating method was utilized to create a crosswalk between scores of the FCT and MMSE. Further, the association of scores of the FCT and MMSE with hippocampal volumes was investigated. There were 241 subjects aged 60 years or above enrolled in this study, including 107 adults with cognitive abilities in normal range, 107 patients with mild cognitive impairment (MCI), and 27 patients with mild Alzheimer disease (AD). The AUC of FCT for detection of cognitive impairment (MCI and mild AD) was 0.885 (95% CI 0.838 to 0.922). The sensitivity and specificity of FCT for the diagnosis of cognitive impairment were 80.6% and 84.11 %, respectively. FCT’s diagnostic performance was superior to that of MMSE in the same cohort. Mean completion time of FCT was 339.9 ± 67.7 seconds (5-6 min). In addition, a conversion table between scores on the FCT and MMSE was created. Further, the FCT scores were positively correlated with hippocampal volumes. The FCT is a novel, reliable, and valid cognitive screening test for the detection of dementia at early stages.

As old population is dramatically growing, the detection of early cognitive deficit will become increasingly crucial. Effective cognitive screening test with quick and convenient merits will ensure recognition of early cognitive deficit and timely intervention [1, 2]. Cognitive screening test should have good sensitivity and specificity for detecting cognitive impairment at the early stage, including mild cognitive impairment (MCI) and mild dementia [3]. Given time pressure in clinical practice or large-scale epidemiological studies, it would be more preferable if a cognitive screening tool can be completed in the minimum time possible. Despite the existence of numerous cognitive screening tools, several limitations need to be remedied [1, 2, 4], including low accuracy for diagnosing mild cognitive deficit [5-7] or a long time consumption [8-10]. Thus, the tradeoff between statistical robustness and administration time should be carefully weighted when novel cognitive screening tool is developed.

According to Jeremy Brown, short cognitive tests can be further categorized into three groups: (1) Short questionnaires, (2) Highly selective tests, and (3) Multi-domain tests [11]. Although short questionnaires or highly selective tests may take less than 5 minutes to administrate [11, 12], multi-domain tests are the most useful tools for capturing deficits in a variety of cognitive domains, but typically at the cost of long administration time (usually > 10 minutes) [11]. Many screening tools are currently available but no tools meet the four important requirements for widespread use in clinical practice or large-scale epidemiological studies — that is, capture a clinically acceptable range of cognitive domains, take short time to administrate (around 5 minutes), have high accuracy for detecting cognitive impairment, and incorporate visual recall, which is the earliest deficits in Alzheimer’s disease (AD) patients [13]. In the present study, we developed a novel cognitive screening tool, Five-minute cognitive test (FCT), to achieve these required qualities.

**Figure 1.**
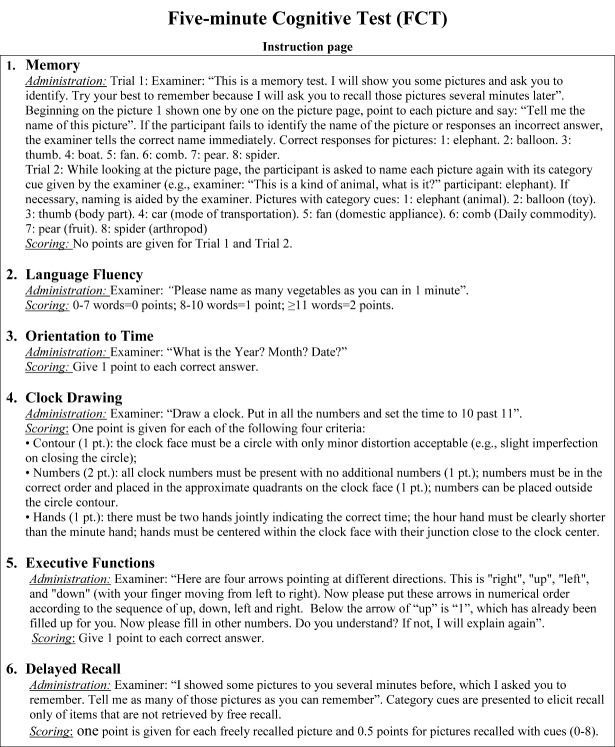
Instruction page of Five-minute Cognitive test.

**Figure 2.**
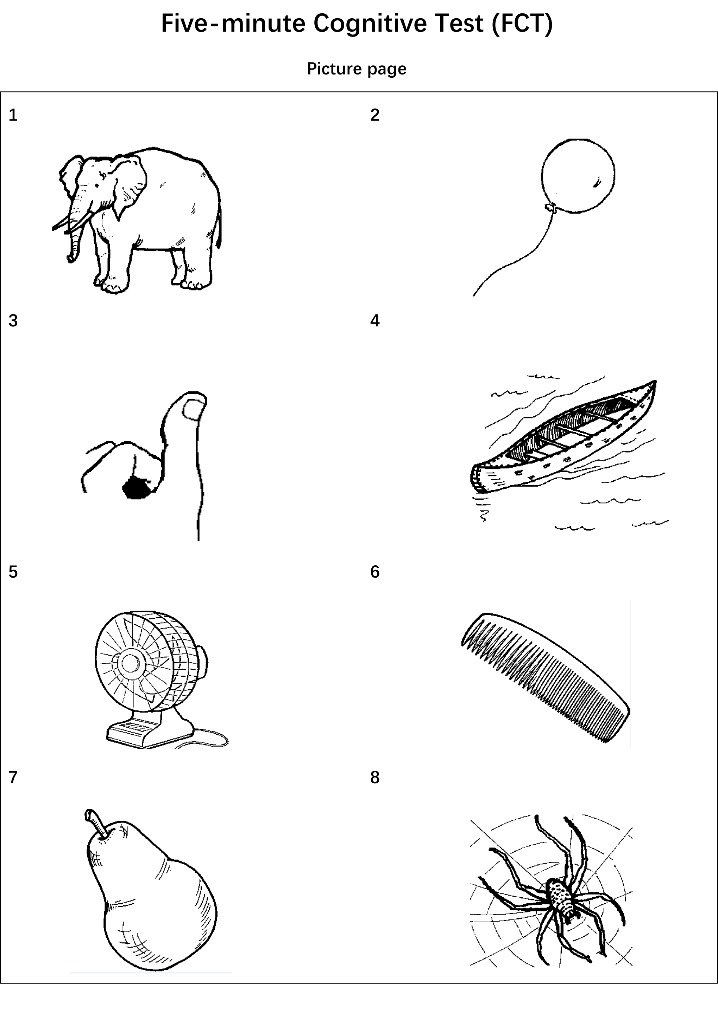
Picture page of Five-minute Cognitive test.

First, we compared the diagnostic performance of FCT and Mini-Mental State Examination (MMSE) for discriminating cognitive ability in normal range from mild cognitive decline. Second, equipercentile equating method was utilized to create a crosswalk between scores of the FCT and MMSE. Third, the relationships between the FCT scores and other neuropsychological assessments were examined. Finally, we investigated the association of the FCT scores with hippocampal volumes in non-demented individuals.

## MATERIALS AND METHODS

The study was approved by the Committee on Medical Ethics of Zhongshan Hospital, Fudan University.

### FCT design

**Figure 3.**
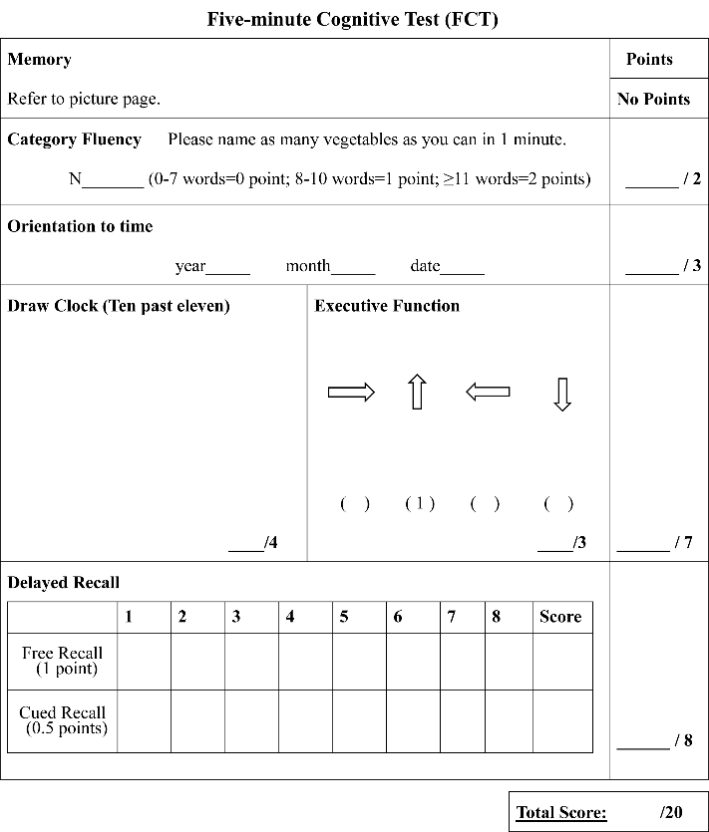
The FCT test.

The FCT was developed in a multiphase study and the final version was established to evaluate five cognitive domains, including episodic memory, language fluency, time orientation, visuospatial function, and executive function (Fig. 1-3). Eight culturally neutral pictures were selected from the International Picture Naming Project as test items of episodic memory. The FCT scores range from 0 to 20 and lower scores reflect worse cognitive performance.

### Subjects

There were 360 subjects aged 60 years or above enrolled in this study, including 226 individuals with cognitive abilities in normal range, 107 patients with MCI and 27 patients with mild AD. Written informed consents were obtained from all participants or authorized representatives.

All subjects had abilities of intact understanding and fluent expression in Mandarin Chinese and received 6 years or more of education. Subjects with any severe neurological diseases but AD and MCI were excluded.

The cognitive ability in normal range was defined as subjects having total 24 scores or above assayed by MMSE [5] and 0 score of Clinical Dementia Rating (CDR)[14]. MCI patients had a score of 24 or higher on the MMSE, a score of 0 or 0.5 on CDR, a subjective cognitive complaint, objective cognitive impairment as examined by a comprehensive battery of neuro-psychological assessments, essentially preserved activities of daily living, and were not demented [3, 15]. The mild AD group met the National Institute of Neurological and Communicative Disorders and Stroke and AD and Related Disorders Association criteria for probable AD [16] and had a score of 0.5 or 1 on the CDR.

### Neuropsychological and clinical assessments

Participants received a battery of cognitive and clinical assessments, including FCT, MMSE [5], Auditory Verbal Learning Test (AVLT)[17], Rey-Osterrieth Complex Figure Test (CFT)[18], Boston Naming Test (BNT-30), Animal Fluency Test (AFT)[19], Trail Making Test A and B (TMT-A, TMT -B)[20], Symbol Digit Modalities Test (SDMT)[21], CDR, Activities of Daily Living scale (ADL), Geriatric Depression Scale (GDS-15)[22]. Each participant with MCI or mild AD received a CT/MRI scanning.

### MRI data acquisition

The data of high-resolution structural MRI were acquired from a 3.0 Tesla Siemens Verio MRI scanner. Participants were scanned using a three-dimensional T1-weighted MPRAGE sequence at the Department of Radiology, Zhongshan Hospital, Fudan University. MRI series included T1-weighted 3D fast, spoiled gradient recalled echo images and other sequences such as T2-weighted and FLAIR images to visualize focal lesions of cortical or white matter. The scanning parameters were as follows: TR/TE: 1900/2.5 ms, flip angle: 9^?^, slice thickness 1 mm, 176 contiguous sagittal slices, field of view 256 mm, voxel resolution 1 × 1 × 1 mm, 8-channels head receiver coil. Each 3-D T1-weighted MPRAGE sequence scan lasted for 4 min and 18 sec. All subjects were right-handed.

### Hippocampal volumes

CAT12 toolbox (C. Gaser, Structural Brain Mapping group, Jena University Hospital, Jena, Germany) implemented in SPM12 (www.fil.ion.ucl.ac.uk) were utilized to perform the voxel-based morphometry (VBM) analysis. The morphometric analysis by regions of interest (ROIs) was applied to extract the data of hippocampal volume. The methods have been described previously [23, 24] and can be found at the CAT12 website (www.neuro.uni-jena.de/cat12/CAT12-Manual.pdf).

**Table 1:** Demographics and clinical characteristics according to diagnosis.

Characteristics	CN (n =107)	MCI (n = 107)	Mild AD (n = 27)	P value
Age, years	69.6 ± 5.4	69.7 ± 5.7	72 ± 7.9	0.149
Male/Female, n	40/67	39/68	10/17	0.99
Education, years	11.7 ± 2.8	11.4 ± 2.9	9.9 ± 4.14 $	0.02
CDR	0 ± 0.05	0.36 ± 0.22 #	0.96 ± 0.1 $, &	<0.001
ADL	20.2 ± 0.4	20.8 ± 0.8 #	24.6 ± 2.9 $, &	<0.001
MMSE	27.9 ± 1.28	26.3 ± 1.9 #	20.1 ± 2.9 $, &	<0.001
FCT	17.8 ± 1.2	14.9 ± 2.8 #	8 ± 3.2 $, &	<0.001
AVLT- delayed recall	5.94 ± 2.05	2.91 ± 2.08	/	<0.001
Animal fluency	16.7 ± 3.63	13.9 ± 3.71	/	<0.001
BNT-30	24.2 ± 3.28	21.7 ± 3.76	/	<0.001
TMT-A	56.1 ± 13.1	72.2 ± 25.4	/	<0.001
TMT-B	153 ± 44	201 ± 59.3	/	<0.001
CFT	30.3 ± 2.92	26.4 ± 4.72	/	<0.001
SDMT	37.9 ± 7.62	30.9 ± 9.33	/	<0.001

Abbreviations: CN: Cognitively normal; MCI: Mild cognitive impairment; AD: Alzheimer’s disease; CDR: Clinical dementia rating scale; ADL: Activities of Daily Living scale; MMSE: Mini-mental state examination; FCT: Five-minute cognitive test. AVLT: Auditory Verbal Learning Test; BNT-30: Boston Naming Test-30; TMT-A: Trail Making Test A; TMT-B: Trail Making Test B; CFT: Rey-Osterrieth Complex Figure Test; SDMT: Symbol Digit Modalities Test.

# that is marked behind “MCI group” represents p < 0.05 when the data from CN group and MCI group were compared.

$ that is marked behind “AD group” represents p < 0.05 when the data from CN group and AD group were compared.

& that is marked behind “AD group” represents p < 0.05 when the data from MCI group and AD group were compared.

**Figure 4.**
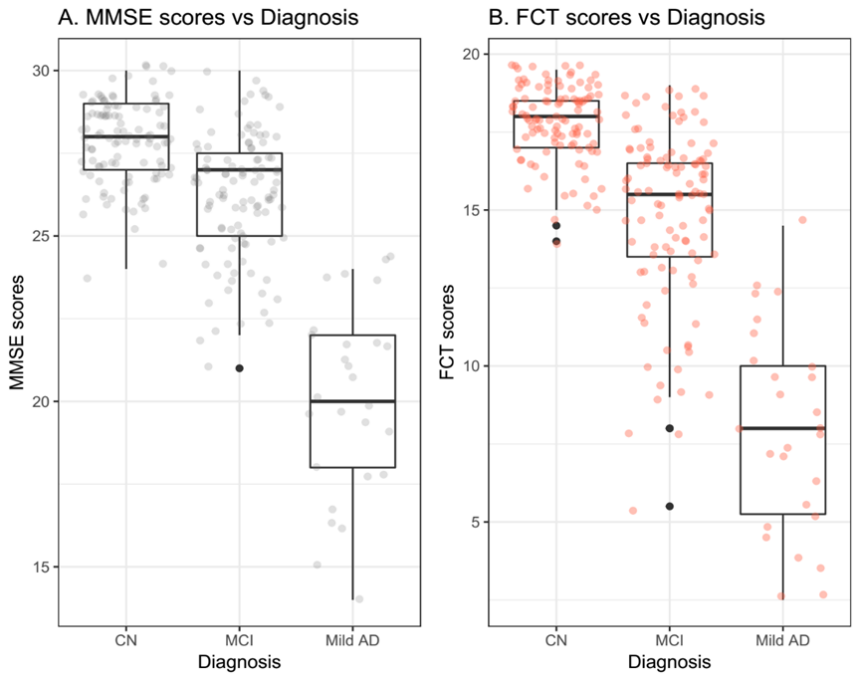
FCT and MMSE scores in three diagnostic groups. Abbreviations: CN: Cognitively normal; MCI: Mild cognitive impairment; AD: Alzheimer’s disease; MMSE: Mini-mental state examination; FCT: Five-minute cognitive test.

### Statistical analysis

The differences in continuous variables between groups were identified using the F-test. The Pearson x^2^ test was used to compare the distributions of categorical variables among healthy controls, patients with MCI and mild AD. Diagnostic performance was examined using the area under the curve (AUC), sensitivity, and specificity. The Intraclass Correlation Coefficient (ICC) was utilized to assess the inter-rater and test-retest reliability. Additionally, FCT scores were equated to MMSE scores using the equipercentile equating method [25, 26], which has been applied to equate a variety of standardized tests[27-29]. All data analyses were conducted with R statistical software (R version 3.3.3). The level of statistical significance was set at p < 0.05.

## RESULTS

### Demographic and clinical information

Initially, there was a total of 360 subjects, including 226 subjects with normal cognition, 107 subjects with MCI and 27 patients with mild AD. However, we found that controls have significantly higher education than subjects with MCI (12 ± 2.9 vs 11.4 ± 2.9; p < 0.001). It is well known that education is a factor that affects the scores of the cognitive assessments. Thus, we applied propensity score matching (PSM) to minimize confounding biases [30, 31]. Through PSM with ratio of 1 to 1 (Controls: MCIs), a total of 107 healthy controls were selected from the initial 226 healthy controls. The demographic and clinical data of 241 participants were listed in Table 1.

### Relationship between FCT scores and demographics

The FCT scores were significantly correlated with age (r = -0.21, p < 0.001) and educational year (r = 0.23, p < 0.001) in the whole sample. There was no significant difference in FCT score between males and females (p > 0.05).

**Figure 5.**
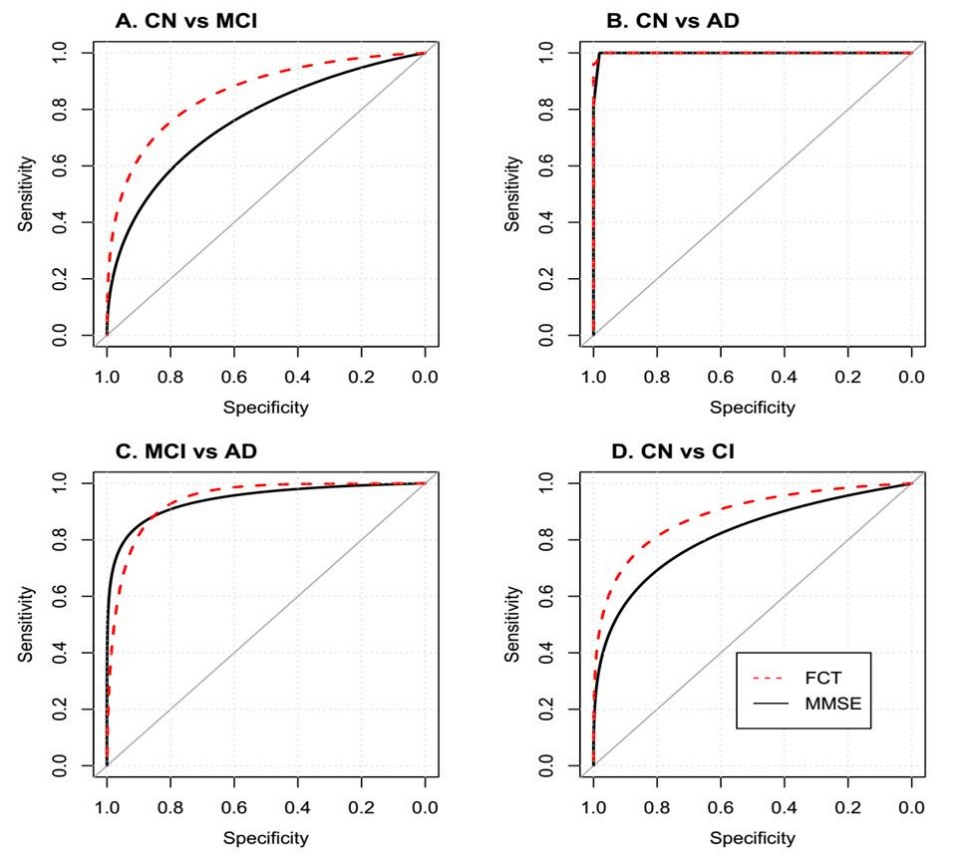
ROC analysis of FCT and MMSE. Abbreviations: CN: Cognitively normal; MCI: Mild cognitive impairment; AD: Alzheimer’s disease; CI: Cognitively impaired; MMSE: Mini-mental state examination; FCT: Five-minute cognitive test.

### The completion time of FCT

The average administration time was 313.3 ± 41.6 s in individuals with cognitive abilities in normal range, 360 ± 61.7 s in patients with MCI, 466.5 ± 96.4 s in patients with mild AD, respectively.

### FCT and MMSE scores in three diagnostic groups

As shown in Table 1 and Figure 4, the average FCT scores were 17.8 ± 1.2 in normal controls, 14.9 ± 2.8 in patients with MCI, and 8 ± 3.2 in patients with mild AD, respectively. The average MMSE scores were 27.9 ± 1.28 in normal controls, 26.3 ± 1.9 in patients with MCI, and 20.1 ± 2.9 in patients with mild AD, respectively.

### Diagnostic performance of FCT and MMSE

For discriminating CN from MCI (Fig. 5A), the AUC of FCT was 0.856 (0.802 - 0.900) with sensitivity of 75.7 % (66.5 % to 83.5 %) and specificity of 84.11 % (75.8% to 90.5 %) when the cut-off point was set to 16.5. The AUC of MMSE was 0.758 (0.695 - 0.814) with sensitivity of 74.8 % (65.4 % to 82.7 %) and specificity of 63.6 % (53.7 % to 72.6 %) when the cut-off point was set to 27.

For discriminating between CN and AD (Fig. 5B), the AUC of FCT was 1 (0.972 - 1) with sensitivity of 100 % (87.2 % to 100 %) and specificity of 98.13 % (93.4% to 99.8% when the cut-off point was set to 14.5. The AUC of MMSE was 0.998 (0.969 - 1) with sensitivity of 100 % (87.2 % to 100 %) and specificity of 98.13 % (93.4 % to 99.8 %) when the cut-off point was set to 24.

**Table 2:** Equivalent MMSE scores are shown for possible scores on the FCT.

FCT	Equivalent MMSE		FCT	Equivalent MMSE		FCT	Equivalent MMSE		FCT	Equivalent MMSE
0	10		5.5	18		11	23		16.5	27
0.5	10		6	19		11.5	24		17	27
1	11		6.5	20		12	24		17.5	28
1.5	12		7	21		12.5	24		18	28
2	12		7.5	21		13	24		18.5	29
2.5	13		8	22		13.5	25		19	29
3	14		8.5	22		14	25		19.5	30
3.5	15		9	22		14.5	25		20	30
4	16		9.5	23		15	26			
4.5	17		10	23		15.5	26			
5	17		10.5	23		16	26			

Abbreviations: MMSE: Mini-mental state examination; FCT: Five-minute cognitive test.

For discriminating MCI from AD (Figure 5.C), the AUC of FCT was 0.942 (0.888 - 0.975) with sensitivity of 96.3 % (81 % to 99.9 %) and specificity 82.24 % (73.7% to 89 %) when the cut-off point was set to 12.5. The AUC of MMSE was 0.97 (0.925 - 0.992) with a sensitivity of 100 % (87.2 % to 100 %) and specificity 83.2 % (74.7 % to 89.7 %) when the cut-off point was set to 24.

For discriminating between CN and CI (MCI + AD; Figure 5.D), the AUC of FCT was 0.885 (0.838 - 0.922) with sensitivity of 80.6 % (72.9 % to 86.9 %) and specificity of 84.11 % (75.8 % to 90.5 %) when the cut-off point was set to 16.5. The AUC of MMSE was 0.806 (0.751 - 0.854) with sensitivity of 59.7 % (50.9 % to 68.1 %) and specificity of 85.1 % (76.9 % to 91.2 %) when the cut-off point was set to 26.

### Inter-rater and test-retest reliability

To evaluate the inter-rater reliability of FCT, the data were collected twice from a subsample of 22 (CN and MCI) by two raters (Zhang and Wang). The average interval was 48.7 ± 20.9 days. Intraclass correlation coefficient (ICC) is 0.91, indicating an excellent inter-rater reliability. Regarding the test-rest reliability, the data were collected twice from another subsample of 16 (CN and MCI) by the same rater (Zhang). The average interval was 64.7±17.7 days. ICC is 0.86, indicating a good test-retest reliability.

### Crosswalk between corresponding scores of the FCT and MMSE

**Figure 6.**
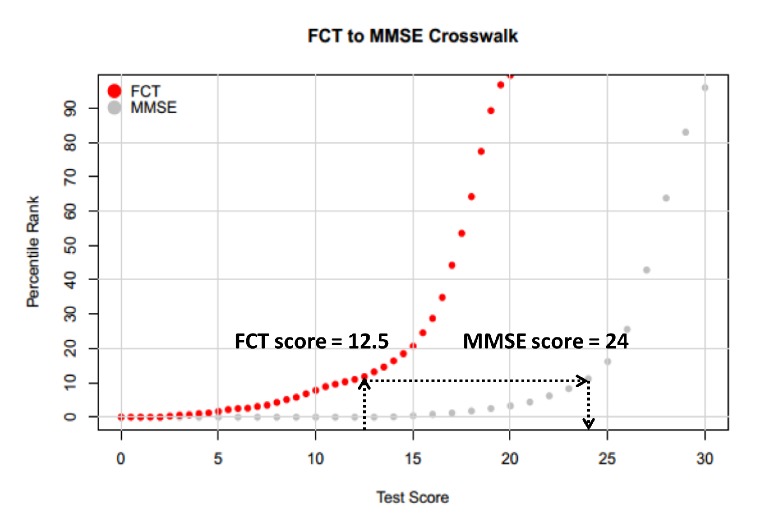
A plot of the equipercentile equivalent scores on the MMSE and FCT. Abbreviations: MMSE: Mini-mental state examination; FCT: Five-minute cognitive test.

The equipercentile equating analysis was utilized and equivalent MMSE scores were shown for possible scores of the FCT (Table 2). In addition, a plot of the equipercentile equivalent scores on FCT and MMSE is demonstrated in Figure 6. For instance, a score of 12.5 on the FCT is equivalent to a score of 24 on the MMSE.

### Correlations between the FCT scores and other neuropsychological assessments

**Figure 7.**
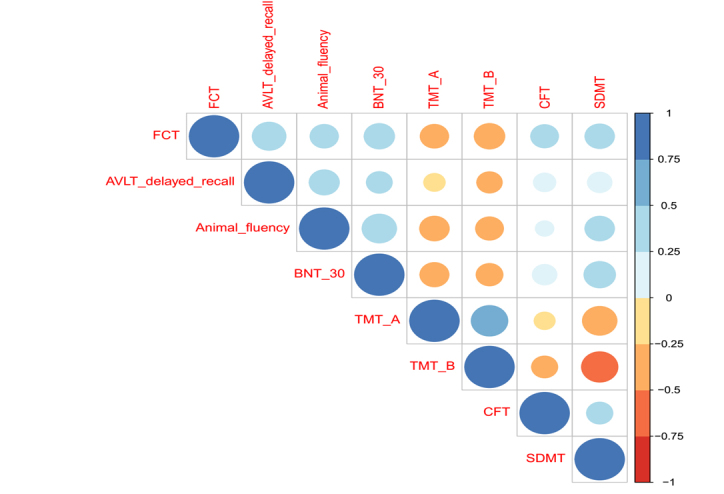
Correlations between the FCT scores and other cognitive domains. Abbreviation: FCT: Five-minute cognitive test. AVLT: Auditory Verbal Learning Test; BNT-30: Boston Naming Test-30; TMT-A: Trail Making Test A; TMT-B: Trail Making Test B; CFT: Rey-Osterrieth Complex Figure Test; SDMT: Symbol Digit Modalities Test.

In these correlational analyses, there was a total of 333 non-demented individuals (226 individuals with normal cognition and 107 individuals with MCI). Spearman’s correlation analyses were utilized to examine the relationships between the FCT scores and other neuropsychological assessments (Figure 7). The FCT scores were positively associated with AVLT delay recall (rho = 0.46, p < 0.001), animal fluency (rho = 0.33, p < 0.001), BNT-30 (rho = 0.38, p < 0.001), CFT (0.32, p < 0.001) and SDMT (rho = 0.36, p < 0.001) scores. Further, the FCT scores were negatively associated with TMT-A (rho = -0.33, p < 0.001) and TMT-B (rho = -0.38, p < 0.001) scores.

### Correlations of the FCT and MMSE scores with hippocampal volumes in non-demented elderly

**Figure 8.**
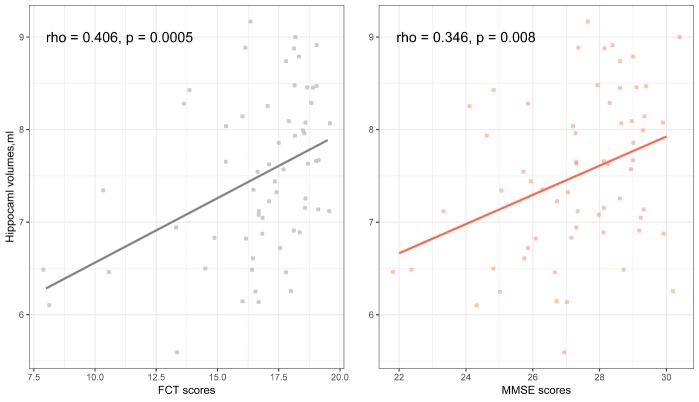
Correlations of FCT and MMSE scores with Hippocampal volumes in non-demented elderly. Abbreviations: MMSE: Mini-mental state examination; FCT: Five-minute cognitive test.

In a subsample of 62 non-demented elderly (including 32 individuals with cognitive abilities in normal range and 30 individuals with MCI) with hippocampal volume data, the correlations of the FCT and MMSE scores with hippocampal volumes were evaluated using the *cocor* package [32]. The results showed that both FCT (rho = 0.406, p = 0.0005) and MMSE (rho = 0.346, p = 0.008) scores were positively correlated with hippocampal volumes while there was no significant difference in the magnitude of two correlations (p > 0.05; Fig. 8).

## DISCUSSION

In this study, we developed a new cognitive screening test that can quickly and accurately identify cognitive deficits. The average administration time of FCT was found to be 339.9 ± 67.7 s (5-6 min) in the whole sample. The validity of FCT for discriminating subjects with cognitive abilities in normal range from mild cognitive impairment (MCI and mild AD) was found to be clinically acceptable and superior to MMSE in sensitivity (80.6% *vs* 59.7%), specificity (84.11 % vs 85.1%), and AUC (0.885 vs 0.806). In addition, equipercentile equating method was used to create a conversion table, which enables easy and direct comparison of scores on FCT and MMSE.

The FCT was designed to capture deficits in a broad range of cognitive domains, including episodic memory, language fluency, time orientation, visuospatial function, and executive function. Episodic memory deficits have been found to predict progression to AD among subjects with MCI [33]. Therefore, the greater emphasis on episodic memory places the FCT at an advantage over the other existing cognitive screening tests for the early detection of AD, the most common type of dementia [34]. In addition, compared to MMSE, the FCT includes more robust measures of executive and visuospatial function, which may contribute to the reduction of practice and ceiling effects.[Table T3-ad-10-6-1258]

**Table 3 T3-ad-10-6-1258:** Demographic and clinical information of 62 non-demented elderly.

Variables	Overall sample (n = 62)
Age, y	69.4 ± 5.6
Female gender, n (%)	45 (72.6)
Education, y	12 ± 2.8
FCT	16.7 ± 2.5
MMSE	27.3 ± 1.9
Hippocampal volume, ml	7.5 ± 0.9

Abbreviations: MMSE: Mini-mental state examination; FCT: Five-minute cognitive test.

Because the MMSE has been extensively used in the cognitive assessment of older people in both clinical research and practice, conversion scores between FCT and MMSE will facilitate the adoption of the FCT. The linkage between FCT scores to MMSE scores is helpful to interpret individual performance and thus facilitate use of this new cognitive screening test. Our study showed that there is a good crosswalk between corresponding scores of the FCT and MMSE. For example, an FCT score of 12.5 is equivalent to a MMSE score of 24 (Fig. 6).

Finally, to examine whether the FCT scores are sensitive to changes in AD-related brain region, Spearman’s correlation test was conducted to investigate the association of the FCT scores with hippocampal volumes in non-demented elderly. Our data found a positive correlation between the FCT scores and hippocampal volumes (r = 0.406, p = 0.0005), which play an important role in episodic memory [35-37] and also correlates to MCI [38] and AD dementia [39]. This may be explained by the fact that the FCT was designed with a heavy emphasis on episodic memory (8 out of 20 points). Further, previous studies reported a specific memory profile in AD patients, which is characterized by a diminished free recall ability that is only marginally ameliorated by cueing [40, 41]. These neuropsychological findings have also been integrated into the development of the FCT. The cued recall technique, utilized in the FCT, can isolate the amnesic syndrome of the medial temporal type [41, 42]. In future studies, it would be very important to determine the exact value of the FCT scores in combination with hippocampal volumes for predicting cognitive decline and conversion from MCI to AD dementia.

The current study has several important clinical implications. First, given the growing prevalence of cognitive impairment, rapid, efficient, and valid cognitive screening tests are essential. However, most of the existing brief cognitive tests were designed for use in western developed countries and therefore pose application problems when applied in Chinese people [43]. Translation into Chinese may help alleviate this issue, although the translation process often contributes to the alterations of the original neuropsychological properties. Thus, brief cognitive tests that transcend language and cultural barriers are needed. In an effort to circumvent the need to translate to other languages, items of FCT were designed to be as culture-neutral as possible. Second, previous studies found that a picture-based memory test can discriminate between normal controls and patients with AD and predict progression from MCI to AD with high sensitivity and specificity [33, 40]. These neuropsychological findings should be integrated into the development of novel screening tools. Thus, for the FCT, the recall of pictures rather than words was used to capture deficit in episodic memory. This design may hopefully facilitate the adoption of the FCT in other non-Chinese speaking countries. Finally, most of popular screening tools take ten minutes or more to complete [5, 8]. The cognitive screening tests with shorter administration time would be more desirable when used in the doctor’s office or large-scale epidemiological studies.

There are several limitations that should be addressed. First, in this study, participants were eligible if they had 6 years or more of education. This limits our ability to generalize our findings to other population with less educational levels. This limitation should be remedied in further studies. Second, because the items of episodic memory were pictured-based, the FCT cannot be administered to subjects with severe visual impairment. The braille version of FCT should be developed in the future. Third, the sample size of the AD group is relatively small. Further studies with larger sample size of AD patients are warranted.

In conclusion, the FCT provides an alternative instrument and is superior to MMSE in discriminating normal cognitive abilities from mild cognitive impairment. Further, there is a good crosswalk between FCT and MMSE scores using equipercentile equating as well as positive correlation between FCT scores and hippocampal volume. These findings may facilitate the adoption of FCT in clinical research and practice.

### Competing interests

Author CZ holds shares of Shanghai Rixin Bitech co, LTD, which dedicates to develop drugs for preventing and treating AD. Other authors declare no competing financial interests.
